# Physical Activity Effects on Blood Parameters, Growth, Carcass, and Meat and Fat Composition of Portuguese Alentejano Pigs

**DOI:** 10.3390/ani11010156

**Published:** 2021-01-12

**Authors:** José Manuel Martins, David Silva, André Albuquerque, José Neves, Rui Charneca, Amadeu Freitas

**Affiliations:** 1 MED—Mediterranean Institute for Agriculture, Environment and Development & Departamento de Zootecnia, Escola de Ciências e Tecnologia, Universidade de Évora, Pólo da Mitra, Ap. 94, 7006-554 Évora, Portugal; jneves@uevora.pt (J.N.); aagbf@uevora.pt (A.F.); 2 Rua José Elias Garcia, 37, 7000-609 Évora, Portugal; davidandredasilva@gmail.com; 3 MED—Mediterranean Institute for Agriculture, Environment and Development, Universidade de Évora, 7006-554 Évora, Portugal; andrealb@uevora.pt; 4 MED & Departamento de Medicina Veterinária, Escola de Ciências e Tecnologia, Universidade de Évora, Pólo da Mitra, Ap. 94, 7006-554 Évora, Portugal; rmcc@uevora.pt

**Keywords:** Alentejano pig, local pigs, physical activity, blood biochemistry, animal performance, meat quality

## Abstract

**Simple Summary:**

Outdoor farming systems are associated with health, animal welfare, sustainability, and food security, making them highly desirable for consumers. In this trial, it was possible to confirm that pig physical activity, a major factor in the traditional and extensive production systems of the Alentejano pig, influences animal welfare and the results of biochemical and zootechnical parameters. In this perspective, breeding pig breeds that perform spontaneous physical activity has greater advantages for producers, as pigs can reach the same slaughter weight in less time and with lower feed costs than animals reared in individual pens. Based on these results, the negative impacts that system intensification (with the consequent reduction in the vital space of each animal and the physical activity performed) may have on animal welfare, animal performance and on the quality of the meat obtained, should be considered.

**Abstract:**

This study evaluated the effects of physical activity on blood, growth, carcass, and meat quality of finishing Alentejano (AL) pigs. Pigs, reared from ~87 to 160 kg in individual pens (no exercise area, NE group, *n* = 9) or in an outdoor park (with exercise area, WE group, *n* = 9), were fed commercial diets (85% ad libitum). WE pigs were in a park (~400 m^2^) with a feeding area separated from the drinking area with an automatic waterer, forcing pigs to walk at least 800 m daily. Blood samples were obtained during the trial (weeks 2, 11, and 18) and at slaughter. The left-side carcass was submitted to commercial cuts, and samples from loin, tenderloin, and dorsal subcutaneous fat (DSF) were analyzed. Plasma urea in WE pigs was higher in weeks 2 and 11, while cholesterol, LDL-cholesterol, cholesterol to HDL-cholesterol and LDL- to HDL-cholesterol ratios, and cortisol were lower in weeks 11 and 18. Final weight and average daily gain were higher and feed conversion ratio lower in WE pigs. DSF thickness and carcass weight were higher in WE pigs, leading to higher carcass yield. Finally, loin and tenderloin from WE pigs presented higher total protein content. These data show that allowing physical activity affects metabolism, growth, and carcass and meat quality of AL pigs.

## 1. Introduction

Alentejano (AL) pig, a local breed from the southern region of Portugal genetically similar to the Iberian pig [1], is traditionally reared outdoors and is well adapted to the environmental conditions. Finished on oak woodland pasture during autumn/winter months, they are slaughtered at heavy body weights (BW) (150 to 160 kg) for the manufacture of high-quality dry-cured products [2]. Contrary to the majority of production pigs, which spend their whole life indoors and confined at high density in pens [3], these pigs have access to outdoor areas, which allow moderate to high physical activity throughout their life. These outdoor systems, alternatives to indoor systems, may become more popular as consumers’ awareness and environmental or animal welfare regulations alter. However, presently, a semi-extensive system based on the production of high-quality fresh meat (“Carne de Porco Alentejano”—Protected Designation of Origin, PDO) throughout the year is being increasingly implemented in Portugal. These pigs are still reared outdoors with access to pastures, but there is an increasing pressure to reduce or eliminate the outdoor component. This would enable farmers to avoid the issues presented by pasture area availability and potentially reduce the duration and cost of the production cycle. Although production cycle optimization is important for local pig breed production chain sustainability, for many breeds, like the AL pig, the products obtained for the (niche) market must respect rearing system and specific certification rules. These are related to their image of naturalness and healthiness and recent attention given to outdoor or free-range rearing systems worldwide [4].

A new generation of consumers choose meat products according to perceived eating quality and (affordable) price but also by the nutritional value and ethical quality of the meat, animal welfare issues, and the degree of impact on the environment caused by the production system [5]. There is a belief that the taste and nutritional value of meat from ecologically or non-intensively produced animals are superior to those of conventionally produced ones [6,7]. Pork quality ultimately depends on multiple interactive effects of genotype, rearing conditions, pre-slaughter handling, and carcass and meat processing (reviewed by [8]). However, little attention has been paid to the possible benefit of outdoor increased pig movement [3], particularly to the relation of pigs’ physical activity to blood parameters, growth and slaughter yield characteristics, which is unclear. From the available studies, some found no differences in performance [9], while others reported physical activity/spontaneous exercise effects on carcass length, meat color, and drip loss [10,11]. To our knowledge, no data are available evaluating the effect of outdoor increased movement/physical activity on these traits from AL pigs.

The present study hypothesized that there would be significant associations between long-term physical activity and welfare, blood parameters, growth, carcass and meat, and fat composition from AL pigs reared in individual pens or outdoors with access to an exercise area. Effects on physiological and carcass data, as well as on the physical–chemical characteristics of m. *Longissimus lumborum*, m. *Psoas major*, and dorsal subcutaneous fat were determined.

## 2. Materials and Methods

### 2.1. Animals, Diets, and Experimental Design

This study was carried out in accordance with the regulations and ethical guidelines set by the Portuguese Animal Nutrition and Welfare Commission (DGAV—Directorate-General for Food and Veterinary, Lisbon, Portugal) following the 2010/63/EU Directive.

Made in collaboration with the Alentejano Breeders Association (ANCPA), this study was designed to simulate as closely as possible the rearing and feeding practices in commercial AL pigs’ farms. Experimental animals were divided into two groups similar in age, gender, and body weight distribution. Eighteen female and male purebred AL pigs, surgically castrated within the 1st week of age, were used at an initial BW of 87.3 ± 0.4 kg (mean ± SEM). These animals, originated from the same farm, had a similar rearing history (e.g., feeding and housing) from birth until the beginning of this trial. Pigs were allocated to an area with open-air shaded individual pens (each with 3 m^2^ of area and concrete floor) with zinc sheds (*n* = 9, 5 female and 4 male pigs, group with no exercise area, NE), and an outdoor park (*n* = 9, 5 female and 4 male pigs, group with exercise area, WE). Individual pens limited the physical activity of the pigs to rising, standing, and some walking steps. The outdoor park consisted of a common area (~400 m^2^) equipped with a battery of individual stalls with a feeder, separated by a corridor from the park with the automatic waterer, forcing pigs to walk at least 800 m daily. In the outdoor park, shade was also provided by a zinc shed (~50 m^2^ of area), but trees were also present. Individual pens and outdoor areas were contiguous, and there was visual but limited physical contact within pigs (neighbors) from the NE group and visual but no physical contact between animals from NE and WE groups. Animals were fed two commercial diets, one during the growth period (until 100 kg BW) and another during the finishing period (100–160 kg BW). Composition of the commercial diets, analyzed by the manufacturer, is presented in Table 1.

Diets were individually offered at 85% estimated ad libitum intake [12] to prevent carcass excess adiposity [4,13] that would penalize the carcass yield at slaughter [13], and in a single daily meal (09:00 h) at a weekly adjusted daily rate. Individual feed consumption varied between 2.4 (beginning of the trial) and 3.8 kg/day (end of the trial). All animals had free access to water (nipple drinkers for NE pigs, inox automatic waterer for WE pigs) and daily diet refusals and spillage were measured. No access to pasture was allowed.

During the 130 days of trial, minimum and maximum temperatures, as well as the relative humidity, were recorded. At weeks 2 and 18 of the trial, longissimus lumborum and subcutaneous backfat thickness at P2 site were measured with a Tringa Linear VET ultrasound scanner fitted with a multi-frequency (5 to 10 MHz) linear probe (Esaote Pie Medical, Maastricht, the Netherlands). Blood samples were also collected from overnight fasted animals at weeks 2, 11, and 18 (the day before delivery of the pigs to the slaughterhouse), using the orbital sinus bleeding technique [14]. Samples were obtained in less than 40 s after containment of the animal in a trunk restraint. A final blood sample collection was done at slaughter, during exsanguination. Plasma was obtained by centrifugation (20 min at 4 °C, and 1500× *g*) (Fiberlite F21-8 × 50y rotor, Sorvall Lynx 4000, Thermo Scientific, Waltham, MA, USA) and frozen (−80 °C) (HFU 686 Basic; Heto, Brondby, Denmark) until analysis.

Pigs were slaughtered at ~160 kg BW after CO_2_ stunning and bleeding at an industrial slaughterhouse (Maporal, Reguengos de Monsaraz, Portugal). Each left side of the carcass was submitted to commercial cuts [15], and the individual weights of the loin, tenderloin, liver and gallbladder were recorded. Finally, longissimus lumborum (loin) and psoas major (tenderloin) muscles, freed from visible fat, as well as dorsal subcutaneous fat (DSF) samples, were collected from the left half carcasses, vacuum-packed, and frozen (−30 °C) for analysis.

### 2.2. Diet, Muscles, and DSF Composition

Diet dry matter (UE 500, Memmert, Schwabach, Germany), total ashes, crude protein (N x 6.25) (Kjeldatherm KB-20, Gerhardt, Bonn, Germany, and Kjeltec Auto 1030 Analyzer, Tecator, Bristol, UK), and neutral and acid detergent fibers were determined as previously described [16]. Total lipids were determined using a Soxtherm automatic apparatus (SE416; Gerhardt, Bonn, Germany), and fatty acids (FA) were determined on a lipid extract obtained following the method of Folch et al. [17].

Moisture was determined in loin, tenderloin, and DSF samples according to the ISO-1442 [18]. Total nitrogen from muscles and fat samples were determined by the Dumas combustion method [method 992.15, 16] in a Leco FP-528 Nitrogen/Protein Determinator (Leco Corp., St. Joseph, MI, USA) and crude protein content calculated (N x 6.25). Total lipids were extracted from muscles by accelerated solvent extraction (ASE 100, Dionex Corporation, Sunnyvale, CA, USA). Samples, mixed with diatomaceous earth (Dionex Corporation, Sunnyvale, CA, USA) were extracted with a mixture of chloroform and methanol (60:40, *v*/*v*) containing 100 mg/L BHT (3,5-di-tert-butyl-4-hydroxytoluene) as an antioxidant. The extraction was performed in 34 mL stainless steel extraction cells, at 100 °C and a nitrogen pressure of 12.4 MPa, in two static extraction cycles of 5 min each. The solvent was removed from the collected extracts under vacuum using a Vacobox B-177 equipped with a vacuum controller B-720, and a rotavapor R-114 attached to a water bath B-480 (Buchi, Flawil, Switzerland). Total neutral lipids were extracted from DSF samples as previously described [19].

Ultimate pH (pH_u_) values were determined 24 h post-mortem in accordance with the ISO-2917 [20] by a pH-meter with a puncture electrode (LoT406-M6-DXK-S7/25, Mettler-Toledo GmbH, Gießen, Germany). Water loss was measured as the sample weight loss due to pressure with a piston for 1 min [21]. Myoglobin content was obtained multiplying the haem pigment concentration, determined according to Hornsey [22], by the factor of 0.026 [23]. Finally, total hydroxyproline was analysed according to Woessner [24] and multiplied by the factor 7.14 to obtain the total collagen content of muscle samples [25].

Measurements of surface colour [26] from raw muscles (after 30 min of blooming) and DSF were recorded with a CR-400 colorimeter (Konica Minolta Sensing Europe B.V., Nieuwegein, The Netherlands) equipped with a D-65 illuminant. CIE (Commission Internationale de l’Éclairage) L* (lightness), a* (redness), and b* (yellowness) values resulted from the average of six random readings across sample surface. Chroma, hue angle [26], and saturation [27] were calculated using the following equations: Chroma (C) = √(a*^2^ + b*^2^); hue angle = tan^−1^(b*/a*); Saturation = C/L*.

### 2.3. Plasma Analyses

Plasma levels of total protein, urea, glucose, triacylglycerols, total cholesterol and LDL- and HDL-cholesterol were determined by enzymatic kits (Roche Diagnostics GmbH, Mannheim, Germany) in an automatic analyzer (Roche Cobas 6000 c501, Tokyo, Japan). Plasma levels of phospholipids were determined by enzymatic kit in an automatic analyzer (UniCel DxC 800, Beckman Coulter, Brea, CA, USA). Finally, plasma cortisol concentrations were determined on an IMMULITE 2000 automated immunoassay analyzer (DPC, Los Angeles, CA, USA) by the Immulite 2000 cortisol assay.

### 2.4. Calculations and Data Analyses

Results are presented as arithmetic means and standard errors (SE). All data were tested for normality by the Shapiro–Wilk test. Statistical analysis was performed by Student’s *t*-test with the IBM SPSS Statistics software (IBM SPSS Statistics for Windows, v24.0, IBM Corp., Armonk, NY, USA). Gender was included as a covariate for data evaluation. For the carcass evaluation data, hot carcass weight was also included as a covariate in the model. Differences were considered significant when *p* < 0.05 and *p*-values between 0.05 and 0.10 were considered trends.

## 3. Results

All pigs remained in good health throughout the experimental period, and no diet refusal or spillage was recorded. Visual observations showed that pigs in NE group remained laying most of the day, while those in the WE group walked and moved frequently in the morning and (late) afternoon, exploring the outdoor area. During the experimental period, average daily mean, maximum, and minimum temperatures, and relative humidity were 21.1, 29.5, 14.1 °C, and 51.4%, respectively.

### 3.1. Fasting Plasma Parameters

Total protein, glucose, triacylglycerols, phospholipids, and HDL-cholesterol were not significantly affected by experimental treatments throughout the trial (Table 2, Table 3 and Table 4).

At the beginning of the second week of trial, fasting plasma parameters analyzed were similar for both experimental treatments, except for urea, which was 19.4% higher in WE than in NE pigs (*p* < 0.001) (Table 2).

At week 11 (Table 3), urea was still 17% higher in WE pigs, but total cholesterol and total cholesterol to HDL-cholesterol ratio were respectively 8.3 (*p* = 0.06) and 11% lower (*p* < 0.05) in WE than in NE pigs. Cortisol level in WE pigs was 42.8% lower than in NE pigs (*p* < 0.05).

Finally, at week 18 (Table 4), fasting plasma levels of cholesterol and LDL-cholesterol were 7.5% and 12.9% lower in WE than in NE pigs (*p* = 0.07 and *p* < 0.05, respectively). As for total cholesterol to HDL-cholesterol and LDL-cholesterol to HDL-cholesterol ratios, they were 10.7% and 16.2% lower (*p* < 0.05 and *p* < 0.01, respectively). Cortisol level remained 41% lower in WE than in NE pigs (*p* < 0.01).

At slaughter, several fasting plasma parameters were significantly different between experimental groups. Triacylglycerols, total cholesterol and LDL-cholesterol were 27.6 (*p* < 0.05), 7.8 (*p* < 0.05), and 10.6% lower (*p* = 0.07) in WE than in NE pigs, respectively (Table 5). Total cholesterol to HDL-cholesterol ratio and LDL-cholesterol to HDL-cholesterol ratio were 7.9 (*p* < 0.05) and 10.8% lower (*p* = 0.07) in WE than in NE pigs. Finally, cortisol level was not affected by experimental treatment.

### 3.2. Productive Parameters, Carcass Characteristics, and Cuts Weight

When compared to NE pigs, WE ones presented 2.3% higher final weight (*p* < 0.05) and 5.7% higher average daily gain (ADG) (*p* < 0.01) with similar daily feed intake (3.1 kg/d, data not shown), leading to a 5.5% lower feed conversion ratio (*p* < 0.01) (Table 6). Loin thickness at 160 kg BW and its increase between 90 and 160 kg, although respectively 3.4% and 8.4% higher in WE than NE pigs, were not significantly different. However, DSF thickness and DSF thickness increase were, respectively, 8.8 and 12.3% higher in WE compared to NE pigs (*p* = 0.08 and *p* < 0.05, respectively). Hot carcass weight and yield were also higher (*p* < 0.05 and *p* = 0.10, respectively) in WE pigs, as well as tenderloin cut weight (+12.7%) (*p* = 0.06). Finally, loin, liver, and gallbladder weights were not affected by experimental treatments.

### 3.3. Tissues Chemical and Physical Composition

The chemical composition of both muscles was only affected by experimental treatments on their total protein content. This content was 6.4% higher (*p* < 0.05) in loin and 14.1% higher (*p* < 0.001) in tenderloin of WE pigs (Table 7 and Table 8, respectively). Total intramuscular lipids, pH_u_, water loss, myoglobin, and muscle surface colour assessment were not significantly different between NE and WE pigs in both muscles. The total collagen content was also not significantly affected by treatment (*p* ≥ 0.05).

Finally, DSF total protein, total intramuscular lipids, and colour assessment were not significantly different (*p* ≥ 0.05) between NE and WE pigs (Table 9).

## 4. Discussion

In outdoor rearing systems, pigs have often a lot of environmentally diverse space, which allow physical activity, expression of investigative behavior, and the potential to forage different feedstuffs complementarily to the commercial feed provided [8,28]. All these factors interact to determine the animal response in terms of growth and meat quality [8]. In fact, meat quality from local breeds can be manipulated through feeding and rearing systems, thereby demonstrating positive genotype x environment interactions [8,29]. The Iberian and AL pigs, perfectly adapted to the environment where they are reared, are good walkers and may walk long distances daily searching for available food [2]. Still, the reduction or suppression of the outdoor factor in Iberian and AL traditional production systems is increasingly present nowadays, to avoid the issues originated by (acorn and pasture) area availability and reduce duration and cost of the production cycle. Besides the dangers of not respecting rearing system and specific certification rules of these local pig production systems, to our knowledge, very few studies concerning the effect of outdoor or free-range physical activity on the final product obtained were made in Iberian [3,30] and none in AL pigs. Hence, the present study was undertaken to evaluate the effect of long-term outdoor physical activity on metabolism, growth, carcass, and meat and fat composition of Portuguese obese AL pigs.

The potential use of biochemical blood parameters at slaughter as predictors of health status, genetic disease resistance, performance traits, and meat quality, has been suggested (e.g., [31,32]). For instance, blood concentrations of glucose, lipoproteins, cholesterol, and triacylglycerols are the result of the uptake and production by lipogenic tissues, and therefore, any diet- or genetic-related changes in their levels may help to shed light on specific lipid metabolic pathways [33]. Still, biochemical blood parameters from growing and finishing pigs of the obese AL breed have been scarcely analysed [34]. In this trial, and with the exception of cortisol, the plasma non-lipid parameters observed in AL pigs between 87 and 160 kg BW were within the normal physiological range observed in growing-finishing pigs of lean European breeds [31,35]. These and the plasma lipid parameters were also similar to the ones previously reported in 100 kg AL pigs [36]. However, during field sampling, urea levels from WE pigs were consistently higher than those from NE, although this difference decreased through the trial (19.4% in week 2, *p* < 0.001; 17% in week 11, *p* < 0.05; 3% in week 18, *p* ≥ 0.05). This difference could be due to a higher skeletal muscle protein catabolism [37] related to increased exercise [38] in WE pigs, due to the increased space available, allowing them to be more active and express investigative behaviour [8,39]. According to visual observations made throughout the experimental period, these actions were more intense until the middle of the trial, when weather was cooler. In fact, from week 1 to 12, the average daily temperature was 19.7 °C, with a maximum temperature higher than 30 °C on 27.6% of the days, while from week 13 to 18, average daily temperature was 23.1 °C, with a maximum temperature higher than 30 °C on 72.3% of the days. This suggests that, despite AL pig rusticity, climatic conditions can play a significant role in its outdoor production system [2,40]. Over time, several plasma lipid parameters became significantly different between experimental groups. On weeks 11 and 18, total cholesterol was lower (*p* = 0.07) in WE when compared to NE pigs, which contributed to a decrease (*p* < 0.05, week 18) in total cholesterol to HDL-cholesterol ratio in those weeks. On week 18, the lower total cholesterol level was mainly due to an LDL-cholesterol reduction in WE pigs (*p* < 0.05), which led to a decrease (*p* < 0.01) in the LDL-cholesterol to HDL-cholesterol ratio. The same tendencies were also observed in week 11, without attaining statistical significance. These data agree with the suggestion that exercise has an effect on cholesterol metabolism by inducing subtle changes in the composition of HDL and LDL [41,42]. In our trial, these changes led to favorable variations in two ratios that are important indicators of vascular risk [43], specifically, a decrease in the total cholesterol to HDL-cholesterol ratio and in the LDL-cholesterol to HDL-cholesterol ratio. Finally, cortisol levels detected in WE pigs were the ones closer to the normal physiological range of lean European breeds. Cortisol levels, governed by the hypothalamic-pituitary-adrenal axis, were consistently lower in WE than in NE pigs (26.2, 42.8, and 41% in weeks 2, 11, and 18, respectively), although only attaining statistical significance in weeks 11 and 18. This suggests that WE pigs were subjected to a lower level of environmental stressors than NE ones [44], since stress results in elevated corticosteroids in the pig, both in the short-term [44,45] and in the long-term [46,47]. On the other hand, individual penning is generally associated with a stress response [45], as observed in our trial. Nevertheless, plasma cortisol level in NE pigs between week 11 and week 18 decreased 19.4% (from 225.1 to 181.5 nmol/L), suggesting some adaptation of these pigs to the rearing conditions.

Between the collection of blood at the farm and at slaughter (by exsanguination), pigs are exposed to stressors during transport and handling, involving physical activity, mental influences, social changes (such as the presence of other, unknown pigs), stunning, sticking, and fasting [46,47]. This is generally accepted as influencing blood biochemistry. In this trial, mean values of glucose, lipid parameters, and cortisol were significantly higher in blood collected at slaughter than in blood collected at the farm (Supplementary Table S1), as previously reported [32,46]. These highly significant differences could have been partially due to differences in blood collection sites (in our case, blood from the orbital sinus vs. a mixture of arterial and venous blood), as suggested by Odink et al. [46]. Another factor that could explain this increase in the values observed in the blood of slaughtered animals could be the shift of water from the plasma to the intra- and intercellular spaces. Since pigs were slaughtered in late summer, heat stress conditions during commercial transport could be related to this water shift [48]. This could be also due to the accumulation of lactate in the muscles, leading to a higher tissue osmolality, thus causing hemoconcentration [32]. Finally, transporting and associated handling impose an acute demand on the energy metabolism of pigs [49]. These are considered pre-slaughter stressors, which increase several parameter levels such as in the case of glucose, triacylglycerols, and cortisol [31,50]. In fact, between the last collection on the farm and the one at slaughter, glucose increased 44.8 (NE pigs) and 36.7% (WE pigs), triacylglycerols increased 157.6 (NE) and 100% (WE), and cortisol increased 127.1 (NE) and 273.2% (WE). Interestingly, cortisol levels between week 18 and slaughter presented a higher increase in WE than in NE pigs. This suggests a response of WE animals to the stressful conditions of slaughter, more important than the one registered in the NE group, whose previous and prolonged contact with conditions of discomfort may have helped them to cope with slaughter confinement and handling. Nevertheless, at slaughter, cortisol levels between NE and WE pigs were not significantly different, suggesting similar stress levels that induced similar adrenalin secretion from the adrenal medulla, causing similar depletion of muscle glycogen and leading to identical pH_u_ values in both muscles from WE and NE pigs (see below).

When comparing outdoor vs. confined/indoor production systems, two confounding factors affect the results obtained: (1) the amount of activity performed by the pigs and (2) the environmental influences. Both can account for the differences observed between systems, but they cannot be easily separated [39]. To try to minimize this issue, in the present trial, pigs were reared in contiguous areas with open-air individual pens and an outdoor park, had access to the same amount of commercial diets (85% ad libitum), water ad libitum, and no access to pasture.

Pigs used in this trial had only 87 kg at 475 days of age. Traditional rearing systems of AL and Iberian pigs are characterized by feed restriction of weaned animals until they reach the fattening period, and those used in our trial were obtained from a producer that followed this procedure. This is due to the fact that in these breeds, good examples of local pigs kept to obtain high-quality meat products, optimisation of growth is not the main productive target because a minimum age is required at slaughter to avoid negative meat quality properties for processing (see [2,51]). On the other hand, the use of restricted feeding in young pigs to induce compensatory growth in adults is a common strategy in these production systems [2]. Overall ADG values observed in our trial were similar to the ones observed in 85% ad libitum fed outdoor reared 100 kg AL [40] and ~171 kg Iberian pigs [3], while lower than the ones reported in ad libitum fed 150 kg AL pigs [34], but the feed conversion ratio was slightly higher [40] to similar [34]. Still, and with the same average daily feed intake (3.1 kg/d), WE pigs grew faster than NE ones (583.1 vs. 551.7 g/day, respectively) and therefore presented a higher final weight and a better feed conversion ratio from week 1 to 18 than NE. However, when compared to the one observed in NE pigs, WE feed conversion ratio was 18.6% lower between week 1 and week 9, and 7.1% higher between week 10 and week 18. This shows that NE pigs were more efficient in gaining weight with the feed consumed during the last part of the trial, suggesting some adaptation of these pigs to rearing conditions, as mentioned above. Growth depends on weather conditions, among other factors [8]. When ambient temperature falls below the lower critical limit of the thermoneutral zone, the pig must produce additional heat, by increasing energy intake and/or diverting more energy from growth to maintenance [52]. In this trial, although minimum temperatures registered were only below the lower critical limit of the thermoneutral zone in two inconsecutive days during spring (first part of the trial, with lighter and more cold-susceptible pigs), they seem to have had a more pronounced effect in NE than in WE pigs. Visual observations of the experimental animals showed that in the first part of the trial, when temperatures were cooler, contrary to NE pigs that were in individual pens, WE ones rested and slept in group, which could partly explain the above-mentioned difference. This suggested higher energy use for thermoregulation by NE pigs agrees with their higher feed conversion ratio between weeks 1 and 9, when compared to the one observed in WE pigs. Cold is also related with increased levels of cortisol [48], which could partly explain the higher cortisol levels of NE pigs. On the other hand, increasing temperatures during the second part of the trial did not have a significant effect on daily feed intake in both experimental treatments, as previously reported in a meta-analysis for restricted- when compared to ad libitum-fed pigs [53]. Meanwhile, evidence supports that stress has deleterious effects on animal behaviour and growth rates [48,54], and in our trial, NE pigs presented the higher plasma cortisol levels and lower ADG and total weight gain during trial. Although depending on weather and feed conditions, environmental enrichment (e.g., more space, straw bedding) generally increases the rate of growth, carcass fat (see below), and can improve meat juiciness and taste due to the increased intramuscular fat [6,8]. However, and contrary to what was observed in this trial, some authors (e.g., [55]) found higher ADG values in confined pigs, suggesting that the physical activity effect on animal growth may depend on several factors, namely on the intensity of the physical activity/exercise.

Heavier WE pigs provided heavier carcasses, with higher DSF thickness, contributing to a higher carcass yield, since in pigs, carcass yield increases with fat carcass content [56]. Interestingly, tenderloin also tended to be heavier (*p* = 0.07) in WE than in NE pigs, but not loin. Both muscles have different insertions: the ilium and sacrum for the longissimus lumborum and the lesser trochanter of the femur for psoas major. Consequently, l. lumborum is primarily considered a support/posture and not a locomotion muscle [57], while p. major is a flexor of the hip [58] and therefore could have been more stimulated by the increased locomotion in WE pigs. The increasing pig spontaneous physical activity induced by the higher available rearing area seems therefore to influence the characteristics of muscles more directly stimulated by exercise.

Loin thickness was not affected by experimental treatments, but DSF thickness was. In fact, DSF thickness at 160 kg and its increase during the trial were higher (*p* = 0.08 and *p* < 0.05, respectively) in WE than in NE pigs, and were similar to the ones observed in exercised and sedentary Iberian pigs at ~171 kg [3,30]. Animals in areas with environmental enrichment such as WE, when compared to NE pigs, generally present increases in carcass fat [6,8] and backfat thickness [8,59]. Stressed animals such as NE pigs, when compared to unstressed ones, consume more energy via an anaerobic pathway when stress hormones are released [60], and elevated blood cortisol levels are associated with less efficient feed conversion [61,62]. Overall, the higher feed conversion ratio observed in NE pigs at the first part of the trial coincided with the higher plasma levels of cortisol, which agrees with the previous suggestion. On the other hand, as previously reported [59], the higher ADG and final weight observed in WE pigs may have contributed to these higher values, since both experimental groups were slaughtered at a common time and not at a common end weight. The higher DSF thickness on WE pigs also suggests that the physical activity was not intense enough to contribute to a significantly increased fat catabolism on these animals.

The literature is not consistent about the effects of physical activity/exercise on meat quality characteristics. Several authors agree that muscle chemical composition is not significantly affected by spontaneous to moderate physical activity in pigs [6,9,28,63], while others report some effects [30,59]. Differences in physical activity levels or exercise training intensity, duration, and/or frequency could partly explain the discrepancies among these studies. In this trial, both loin and tenderloin composition were significantly affected by experimental treatments only in their total protein content. WE pigs presented higher total protein content than NE ones in both muscles, suggesting a higher anabolism and/or lower protein catabolism than the latter. In fact, increased protein synthesis is related with increased muscle activity, while increased protein catabolism is associated to decreased muscle activity [64]. This was observed in our trial and also in tenderloins from sedentary Iberian pigs when compared to exercised ones, due to a lower muscle cathepsin activity in the latter [3]. On the other hand, hormones are known to modify the rates of protein turnover in skeletal and cardiac muscle [64]. Contrary to the anabolic signals of GH, IGF-1 and insulin, cortisol is a catabolic glucocorticoid and has an inhibitory effect on protein synthesis [48,65]. This is supported by the higher plasma cortisol during the trial and lower total protein content of both muscles observed in NE pigs. Meanwhile, the theory that high cortisol levels on the long-term basis increase body fatness [66] was not supported by the data from this trial, but visceral fat was not determined.

Influence of physical activity or outdoor rearing on muscle lipid composition seems to be related to the actual rearing conditions of animals (climate, feeding level) [8]. In fact, authors report both similar [3,30,67] or decreased [39,68] muscle lipid contents for moderately exercised/outdoor- compared with indoor-reared pigs. In our trial, with animals in the same climatic and feeding conditions, physical activity did not significantly influenced loin and tenderloin intramuscular fat content, contrary to what was observed in DSF thickness, as previously reported [30]. The constantly lower plasma triacylglycerol levels in WE when compared to NE pigs (17.2%, 6.8%, and 27.6% in weeks 11, 18 and at slaughter) suggest that they were drawn at higher levels from blood by muscle cells for oxidation in WE pigs. This agrees with the suggestion that lipid metabolism in muscle and adipose tissues are differently regulated [69]. Nevertheless, intramuscular fat values observed in both muscles were above 2.5 g/100 g, the threshold below which some meat quality characteristics such as juiciness, taste, and flavour may be negatively affected [70,71]. Finally, the pH_u_ values were close to the higher value of the normal range in pork, which varies between 5.5 and 5.8 [72], and were not affected by experimental treatments. The pH_u_ is related to several meat quality parameters, including L* value and water loss, which were also not affected by experimental treatments in both muscles.

## 5. Conclusions

The effects of physical activity or outdoor rearing of pigs are not consistent. This is possibly due to the use of different breeds together with differences in feeding allowance and composition, and duration and/or intensity of the physical activity. Still, in the present study, it can be concluded that the outdoor physical activity performed by WE pigs affected blood biochemistry, performance, carcass, and meat quality traits in a positive way.

When compared to NE pigs, WE presented during the trial lower blood total cholesterol, LDL-cholesterol, and total cholesterol to HDL-cholesterol and LDL-cholesterol to HDL-cholesterol ratios, and higher HDL-cholesterol. Based on cortisol levels it can also be assumed that these pigs were also less stressed than NE ones. Finally, it was observed that physical activity of AL pigs leads to faster growth with an overall better feed conversion ratio and heavier carcasses with higher carcass yield and DSF thickness. The meat contained more crude protein, and similar IMF and total collagen than that from pigs reared in individual pens. Overall, when analyzing data from pigs reared outdoor and in individual pens, with a reduction in their vital space and physical activity allowance, we have detected effects on animal welfare, animal performance, and the physical-chemical composition of the meat obtained, but not on the DSF. Therefore, based on the present assessment, the reduction or absence of physical activity/spontaneous exercise should be carefully considered before being implemented, namely because of their effects on animal stress.

## Figures and Tables

**Table 1:** Ingredients, chemical and main fatty acid composition of the experimental diets.

	Growing Diet (87–100 kg BW)	Finishing Diet (100–160 kg BW)
Ingredients (g/100 g):		
Barley	15.0	40.0
Wheat	33.1	28.6
Maize	20.0	20.0
Wheat bran	9.0	
Sunflower meal	8.6	5.9
Rapeseed meal	8.5	
Fat	2.7	2.2
Calcium carbonate	1.12	1.4
Dicalcium phosphate	0.67	0.54
Sodium chloride	0.4	0.4
L-lysine	0.37	0.36
DL-methionine		0.07
L-threonine	0.05	0.01
Vitamin and mineral premix ^1^	0.5	0.5
Chemical composition ^2^ (g/100 g DM):		
Dry matter (DM) (g/100 g)	89.3	89.2
Total ashes	5.1	4.6
Crude protein (N x 6.25)	14.0	10.5
Lysine	0.81	0.62
Neutral detergent fibre (NDF)	17.01	14.97
Acid detergent fibre (ADF)	6.80	5.40
Total lipids	4.87	4.25
Palmitic acid (16:0)	0.81	0.71
Stearic acid (18:0)	0.40	0.33
Oleic acid (18:1 *n*-9)	1.42	1.15
Linoleic acid (18:2 *n*-6)	1.15	1.09
∑ Saturated FA (SFA)	1.27	1.09
∑ Unsaturated FA (UFA)	3.01	2.60
Ratio ∑ Unsaturated FA: ∑ SFA	2.37	2.39
Digestible energy (MJ/kg)	13.51	13.48

^1^ Supplied per kg diet fed: vitamin A (E672), 7500 IU; vitamin D3 (E671), 1000 IU; Mn (E5), 60.0 mg; Zn (E6), 110.0 mg; Cu (E4), 10.0 mg; Fe (E1), 135.0 mg; I (E2), 0.8 mg Se (E8), 0.2 mg; Butylated hydroxytoluene (BHT) (E321), 6.6 mg; Ethoxyquin (E324), 1.0 mg; Butylated hydroxyanisole (BHA) (E320), 0.6 mg; 6-Phytase (EC 3.1.3.26), 500 FTU; ^2^ Chemical composition of the commercial diets was analyzed by the manufacturer.

**Table 2:** Plasma parameters from Alentejano pigs kept on individual pens without exercise area (NE, *n* = 9) or outdoors with exercise area (WE, *n* = 9) at week 2 of the trial.

Traits	NE		WE		ANOVA
	Mean	SE	Mean	SE	
Total protein (g/L)	76.9	1.1	76.2	0.8	NS
Urea (mmol/L)	4.58	0.11	5.47	0.11	***
Glucose (mmol/L)	4.04	0.09	3.89	0.06	NS
Triacylglycerols (mmol/L)	0.47	0.02	0.44	0.03	NS
Phospholipids (mmol/L)	1.69	0.06	1.69	0.07	NS
Total cholesterol (mmol/L)	2.80	0.04	2.76	0.06	NS
LDL-cholesterol (mmol/L)	1.39	0.05	1.33	0.04	NS
HDL-cholesterol (mmol/L)	1.40	0.01	1.38	0.01	NS
Total cholesterol: HDL-cholesterol ratio	1.99	0.03	2.02	0.07	NS
LDL-cholesterol: HDL-cholesterol ratio	0.99	0.03	0.97	0.04	NS
Cortisol (nmol/L)	201.5	27.1	148.7	25.6	NS

ANOVA: *** *p* < 0.001; NS, not significant (*p* ≥ 0.05).

**Table 3:** Plasma parameters from Alentejano pigs kept on individual pens without exercise area (NE, *n* = 9) or outdoors with exercise area (WE, *n* = 9) at week 11 of the trial.

Traits	NE		WE		ANOVA
	Mean	SE	Mean	SE	
Total protein (g/L)	71.8	1.0	70.9	1.4	NS
Urea (mmol/L)	3.58	0.12	4.19	0.22	*
Glucose (mmol/L)	4.55	0.16	4.54	0.16	NS
Triacylglycerols (mmol/L)	0.58	0.07	0.48	0.04	NS
Phospholipids (mmol/L)	2.33	0.09	2.29	0.14	NS
Total cholesterol (mmol/L)	3.26	0.12	2.99	0.07	0.06
LDL-cholesterol (mmol/L)	1.49	0.02	1.44	0.07	NS
HDL-cholesterol (mmol/L)	1.49	0.03	1.54	0.02	NS
Total cholesterol: HDL-cholesterol ratio	2.19	0.08	1.95	0.07	*
LDL-cholesterol: HDL-cholesterol ratio	1.00	0.01	0.94	0.05	NS
Cortisol (nmol/L)	225.1	32.0	128.7	21.1	*

ANOVA: * *p* < 0.05; NS, not significant (*p* ≥ 0.05).

**Table 4:** Plasma parameters from Alentejano pigs kept on individual pens without exercise area (NE, *n* = 9) or outdoors with exercise area (WE, *n* = 9) at week 18 of the trial.

Traits	NE		WE		ANOVA
	Mean	SE	Mean	SE	
Total protein (g/L)	73.4	0.8	73.7	0.6	NS
Urea (mmol/L)	4.37	0.08	4.50	0.17	NS
Glucose (mmol/L)	4.17	0.12	4.25	0.11	NS
Triacylglycerols (mmol/L)	0.59	0.05	0.55	0.04	NS
Phospholipids (mmol/L)	1.87	0.04	1.77	0.08	NS
Total cholesterol (mmol/L)	2.92	0.07	2.70	0.08	0.07
LDL-cholesterol (mmol/L)	1.40	0.05	1.22	0.03	*
HDL-cholesterol (mmol/L)	1.42	0.01	1.48	0.03	NS
Total cholesterol: HDL-cholesterol ratio	2.06	0.06	1.84	0.05	*
LDL-cholesterol: HDL-cholesterol ratio	0.99	0.04	0.83	0.03	**
Cortisol (nmol/L)	181.5	22.3	107.0	9.0	**

ANOVA: ** *p* < 0.01; * *p* < 0.05; NS, not significant (*p* ≥ 0.05).

**Table 5:** Plasma parameters from Alentejano pigs kept on individual pens without exercise area (NE, *n* = 9) or outdoors with exercise area (WE, *n* = 9) at slaughter at ~160 kg BW.

Traits	NE		WE		ANOVA
	Mean	SE	Mean	SE	
Total protein (g/L)	75.2	1.1	74.9	1.3	NS
Urea (mmol/L)	4.32	0.20	4.85	0.22	NS
Glucose (mmol/L)	6.04	0.20	5.81	0.11	NS
Triacylglycerols (mmol/L)	1.52	0.15	1.10	0.12	*
Phospholipids (mmol/L)	2.07	0.05	2.06	0.10	NS
Total cholesterol (mmol/L)	3.22	0.07	2.97	0.09	*
LDL-cholesterol (mmol/L)	1.51	0.07	1.35	0.04	0.07
HDL-cholesterol (mmol/L)	1.48	0.02	1.49	0.05	NS
Total cholesterol: HDL-cholesterol ratio	2.16	0.05	1.99	0.04	*
LDL-cholesterol: HDL-cholesterol ratio	1.02	0.04	0.91	0.03	0.07
Cortisol (nmol/L)	412.2	36.5	399.3	36.4	NS

ANOVA: * *p* < 0.05; NS, not significant (*p* ≥ 0.05).

**Table 6:** Growth data and carcass, organ and cuts characteristics from Alentejano pigs kept on individual pens without exercise area (NE, *n* = 9) or outdoors with exercise area (WE, *n* = 9) from ~87 to 160 kg BW.

Traits	NE		WE		ANOVA
	Mean	SE	Mean	SE	
Initial age (d)	475.6	3.1	476.8	2.1	NS
Initial weight (kg)	87.4	0.6	87.1	0.5	NS
Final weight (kg)	159.7	1.0	163.4	1.0	*
Total weight gain (kg)	72.3	0.9	76.4	0.7	**
Average daily gain (g/d) (week 1–week 18)	551.7	7.2	583.1	5.8	**
Average daily gain (week 1–week 9) (g/d)	426.2	16.8	519.8	13.9	***
Average daily gain (week 10–week 18) (g/d)	695.8	11.7	655.7	9.3	*
Feed conversion ratio (week 1–week 18)	5.66	0.07	5.35	0.05	**
Feed conversion ratio (week 1–week 9)	5.70	0.23	4.64	0.13	***
Feed conversion ratio (week 10–week 18)	5.60	0.09	6.00	0.08	*
L. lumborum thickness at 160 kg BW (cm) ^1^	3.81	0.07	3.94	0.06	NS
L. lumborum thickness increase (90–160 kg) (cm) ^1^	0.98	0.06	1.06	0.07	NS
DSF thickness at 160 kg BW (cm)^1^	4.34	0.17	4.72	0.12	0.08
DSF thickness increase (90–160 kg) (cm) ^1^	2.76	0.11	3.10	0.07	*
Hot carcass weight (kg)	124.9	1.2	129.4	1.4	*
Hot carcass yield (%)	78.2	0.3	79.1	0.4	0.10
Loin (kg)	2.00	0.08	2.05	0.07	NS
Tenderloin (g)	235.6	8.0	265.6	13.4	0.06
Liver (kg)	1.80	0.05	1.75	0.04	NS
Gallbladder (g)	65.0	6.2	62.4	4.6	NS

ANOVA: *** *p* < 0.001; ** *p* < 0.01; * *p* < 0.05; NS, not significant (*p* ≥ 0.05); DSF—Dorsal subcutaneous fat; ^1^ Measurements taken at P2 site.

**Table 7:** Physical-chemical composition of longissimus lumborum from Alentejano pigs kept on individual pens without exercise area (NE, *n* = 9) or outdoors with exercise area (WE, *n* = 9) from ~87 to 160 kg BW.

Traits	NE		WE		ANOVA
	Mean	SE	Mean	SE	
Moisture (g/100 g)	70.7	0.5	71.0	0.4	NS
Total protein (g/100 g)	20.4	0.4	21.7	0.5	*
Total intramuscular fat (g/100 g)	7.0	0.3	7.0	0.2	NS
Ultimate pH (24 h)	5.7	0.1	5.7	0.1	NS
Water loss (%)	23.8	1.1	22.3	0.8	NS
Myoglobin (mg/g)	1.44	0.12	1.59	0.09	NS
Total collagen (mg/g DM)	11.4	0.4	10.7	0.3	NS
Lightness (CIE L*)	47.9	0.6	49.4	1.1	NS
Redness (CIE a*)	15.4	0.4	15.2	0.4	NS
Yellowness (CIE b*)	8.3	0.4	8.7	0.3	NS
Chroma (C) ^1^	17.5	0.4	17.5	0.5	NS
Hue angle (H◦) ^2^	28.3	1.0	29.9	0.7	NS
Saturation ^3^	0.37	0.01	0.36	0.01	NS

ANOVA: * *p* < 0.05; NS, not significant (*p* ≥ 0.05); CIE—Commission Internationale de l’Éclairage; ^1^ Chroma (C) = √(a*^2^ + b*^2^); ^2^ Hue angle (H◦) = tan^−1^(b*/a*); ^3^ Saturation = C/L*.

**Table 8:** Physical-chemical composition of psoas major from Alentejano pigs kept on individual pens without exercise area (NE, *n* = 9) or outdoors with exercise area (WE, *n* = 9) from ~87 to 160 kg BW.

Traits	NE		WE		ANOVA
	Mean	SE	Mean	SE	
Moisture (g/100 g)	73.6	0.4	73.4	0.4	NS
Total protein (g/100 g)	19.1	0.4	21.8	0.5	***
Total intramuscular fat (g/100 g)	5.6	0.3	6.1	0.2	NS
Ultimate pH (24 h)	5.8	0.1	5.7	0.1	NS
Water loss (%)	26.5	1.3	26.9	0.9	NS
Myoglobin (mg/g)	4.16	0.20	4.13	0.17	NS
Total collagen (mg/g DM)	9.2	0.6	8.7	0.4	NS
Lightness (CIE L*)	37.7	1.0	37.7	1.0	NS
Redness (CIE a*)	19.4	0.5	18.9	0.9	NS
Yellowness (CIE b*)	8.3	0.5	7.5	1.0	NS
Chroma (C) ^1^	21.1	0.6	20.5	1.2	NS
Hue angle (H◦) ^2^	23.0	0.9	20.6	2.0	NS
Saturation ^3^	0.56	0.02	0.54	0.02	NS

ANOVA: *** *p* < 0.001; NS, not significant (*p* ≥ 0.05); CIE—Commission Internationale de l’Éclairage; ^1^ Chroma (C) = √(a*^2^ + b*^2^); ^2^ Hue angle (H◦) = tan^−1^(b*/a*); ^3^ Saturation = C/L*.

**Table 9:** Physical-chemical composition of dorsal subcutaneous fat from Alentejano pigs kept on individual pens without exercise area (NE, *n* = 9) or outdoors with exercise area (WE, *n* = 9) from ~87 to 160 kg BW.

Traits	NE		WE		ANOVA
	Mean	SE	Mean	SE	
Moisture (g/100 g)	5.3	0.2	5.0	0.2	NS
Total protein (g/100 g)	0.97	0.04	0.90	0.03	NS
Total IM lipids (g/100 g)	93.8	0.3	94.0	0.2	NS
Lightness (CIE L*)	80.4	0.5	80.3	0.6	NS
Redness (CIE a*)	4.34	0.42	4.34	0.35	NS
Yellowness (CIE b*)	4.97	0.29	4.86	0.33	NS
Chroma (C) ^1^	6.6	0.5	6.5	0.5	NS
Hue angle (H◦) ^2^	49.5	1.6	48.4	1.2	NS
Saturation ^3^	0.08	0.01	0.08	0.01	NS

ANOVA: NS, not significant (*p* ≥ 0.05); CIE—Commission Internationale de l’Éclairage; ^1^ Chroma (C) = √(a*^2^ + b*^2^); ^2^ Hue angle (H◦) = tan^−1^(b*/a*); ^3^ Saturation = C/L*.

## Data Availability

Data will not be shared, due to privacy issues.

## Supplementary Materials

The following are available online at https://www.mdpi.com/2076-2615/11/1/156/s1, Table S1: Plasma parameters at week 18 and at slaughter from Alentejano pigs kept on individual pens without exercise area (NE, *n* = 9) or outdoors with exercise area (WE, *n* = 9) from ~87 to 160 kg BW.
