# Supplementary material for: Physical Activity Effects on Blood Parameters, Growth, Carcass, and Meat and Fat Composition of Portuguese Alentejano Pigs
Source: Animals (Basel). 2021 Jan 12;11(1):156. doi: 10.3390/ani11010156 (PMC7827948; doi:10.3390/ani11010156)
Supplement: Supplementary file 1 [file animals-11-00156-s001.pdf]

Supplementary Materials: Physical Activity Effects on Blood Parameters, Growth, Carcass, and Meat and Fat Composition of Portuguese Alentejano pigs

Table S1. Plasma parameters at week 18 and at slaughter from Alentejano pigs kept on individual pens without exercise area (NE, *n* = 9) or outdoors with exercise area (WE, *n* = 9) from ~87 to 160 kg BW.

Traits	Blood Collection at Week 18				Blood Collection at Slaughter				ANOVA		
	NE		WE		NE		WE				
	Mean	SE	Mean	SE	Mean	SE	Mean	SE	G	D	G x D
Total protein (g/L)	73.4	0.8	73.7	0.6	75.2	1.1	74.9	1.3	NS	NS	NS
Urea (mmol/L)	4.37	0.08	4.50	0.17	4.32	0.20	4.85	0.22	0.08	NS	NS
Glucose (mmol/L)	4.17	0.12	4.25	0.11	6.04	0.20	5.81	0.11	NS	***	NS
Triacylglycerols (mmol/L)	0.59	0.05	0.55	0.04	1.52	0.15	1.10	0.12	*	***	NS
Phospholipids (mmol/L)	1.87	0.04	1.77	0.08	2.07	0.05	2.06	0.10	NS	**	NS
Total cholesterol (mmol/L)	2.92	0.07	2.70	0.08	3.22	0.07	2.97	0.09	**	**	NS
LDL-cholesterol (mmol/L)	1.40	0.05	1.22	0.03	1.51	0.07	1.35	0.04	**	0.06	NS
HDL-cholesterol (mmol/L)	1.42	0.01	1.48	0.03	1.48	0.02	1.49	0.05	NS	NS	NS
Total cholesterol : HDL-cholesterol ratio	2.06	0.06	1.84	0.05	2.16	0.05	1.99	0.04	***	*	NS
LDL-cholesterol : HDL-cholesterol ratio	0.99	0.04	0.83	0.03	1.02	0.04	0.91	0.03	**	NS	NS
Cortisol (nmol/L)	181.5	22.3	107.0	9.0	412.2	36.5	399.3	36.4	NS	***	NS

ANOVA: G – Experimental group; D – Collection date; ***$p < 0.001$; **$p < 0.01$; *$p < 0.05$; NS, not significant ($p \geq 0.05$).
